# A new family of bacterial actin-like proteins regulates cell morphology in a filamentous cyanobacterium

**DOI:** 10.1128/msphere.00499-25

**Published:** 2025-10-31

**Authors:** Alicia Nguyen, Garrett M. Jenkins, Peyton D. Brones, Gabriel A. Parrett, Guy M. Hagen, Jeremy M. Bono, Douglas D. Risser

**Affiliations:** 1 Department of Biology, University of Colorado Colorado Springs 14676, Colorado Springs, Colorado, USA; 2 BioFrontiers Center, University of Colorado Colorado Springs 661305, Colorado Springs, Colorado, USA; University of Wyoming, Laramie, Wyoming, USA

**Keywords:** cyanobacteria, actin, bacterial actin-like proteins, cell morphology, phenotypic plasticity

## Abstract

Actin proteins are common to all domains of life and exhibit ATP-dependent polymerization to form filaments. In bacteria, four families of bacterial actin-like proteins (BALPs) have been identified and characterized. These BALPs are involved in plasmid partitioning (ParM), cell division (FtsA), magnetosome positioning (MamK), and cell morphology (MreB). Here, we report the identification of a fifth family of BALP, FcmB. Using the model filamentous cyanobacterium *Nostoc punctiforme*, we demonstrate that FcmB is a BALP that regulates cell morphology in filamentous cyanobacteria. Deletion of *fcmB*, or *fcmC*, which encodes an FcmB-interacting protein, resulted in the loss of rod morphology, similar to the phenotype reported for *mreB* mutants in other bacteria, including cyanobacteria. However, despite the apparent functional similarity, *fcmB* is not a paralog of *mreB*, but rather was acquired by horizontal gene transfer of a plasmid partitioning system and subsequent integration into the chromosome. Fluorescent protein fusions and immunofluorescence demonstrate that FcmB forms membrane-bound filaments which wrap around the circumference of the cell, while FcmC is localized to discrete membrane-associated foci and is essential for proper membrane localization of FcmB. Protein-protein interactions were detected between FcmB and FcmC, but not MreB, indicating that FcmB and MreB do not form heterofilaments. It is currently unclear how FcmBC exerts its effect on cell morphology, but both *mreB* and *fcmB* are ubiquitous in the developmentally complex heterocyst-forming filamentous cyanobacteria, and the presence of two discrete systems modulating cell morphology may be critical for the remarkable degree of phenotypic plasticity observed in these organisms.

**IMPORTANCE:**

Filament-forming actin proteins are found in nearly all living organisms. In bacteria, four families of actin proteins have been defined, with biological functions in plasmid partitioning, cell division, magnetosome positioning, and cell morphology. Here, we identify and characterize FcmB, a fifth family of bacterial actin proteins found in filamentous cyanobacteria, and demonstrate that this family evolved from plasmid partitioning actins but influences cell morphology rather than DNA segregation. Filamentous cyanobacteria exhibit substantial phenotypic plasticity and typically contain both FcmB and MreB, the other actin family known to regulate cell morphology. The presence of two distinct families of actin proteins influencing cell morphology may play a critical role in the ability of these organisms to rapidly alter their cell shape.

## INTRODUCTION

Actin proteins are ubiquitous in all domains of life (1, 2). Although the sequences of bacterial actin-like proteins (BALPs) are highly divergent from eukaryotic actin, BALPs adopt a similar structure and share a basic common function, polymerization to form filaments in an ATP-dependent manner (2). While the primary structure of eukaryotic actin is highly conserved and its role in the eukaryotic cytoskeleton is well defined, several divergent families of BALPs have been characterized in recent years, and unlike eukaryotic actin, these serve diverse functions in the bacterial cell (2) (Table S1). Plasmid partitioning actins, such as ParM, are typically encoded on their associated plasmids and form filaments that are capped at the ends by ParR (3). ParR then binds to a recognition sequence, *parC*, on the corresponding plasmid, and thus as the ParM filaments grow, plasmids are pushed to opposite ends of the cell (3). The MamK-type actins form filaments that direct the positioning of magnetosomes (4). The FtsA family facilitates cell division via the formation of filaments that ring the mid-cell and interact with other divisome components (5). Finally, the MreB actins promote rod-shaped morphology (6). MreB forms membrane-associated filaments along the length of the cell, which directly interact with RodZ to facilitate the formation of rod complexes that include MreC, MreD, RodZ, RodA, and a class B penicillin-binding protein (bPBP) (6). RodA and bPBP subsequently catalyze the synthesis of a new cell wall at these restricted locations, resulting in cell elongation and thus rod-shaped morphology (6).

In the photosynthetic cyanobacteria, homologs of most characterized BALP families, including FtsA, are absent, except for MreB, which is nearly ubiquitous, as are other rod-complex components (7). However, RodZ is conspicuously absent from the cyanobacterial phylum, as BLAST searches (8) using *Escherichia coli* RodZ for query only identify five cyanobacterial species containing RodZ homologs, most of which have low sequence identity. How the cyanobacterial rod complex can function in the absence of RodZ is currently unknown. The near ubiquity of MreB is surprising, considering its highly conserved role in producing rod-shaped cell morphology in other bacteria, coupled with the fact that many MreB-containing cyanobacteria do not display rod-shaped cell morphology (7). However, genetic studies have confirmed that in those cyanobacteria that do display rod morphology, disruption of *mreB* produces a more coccoid cell shape (9–11).

Moreover, many strains of filamentous cyanobacteria exhibit substantial morphological plasticity and can transition between more coccoid and rod-shaped cell morphologies depending on environmental cues and developmental programs. Two well-studied examples of this are complementary chromatic acclimation (CCA) in *Fremyella diplosiphon* (12) and hormogonium development in *Nostoc punctiforme* (13). In *F. diplosiphon*, changes in light quality trigger CCA, which results in both an alteration in the abundance of different photosynthetic pigments, as well as a transition between rod and coccoid cell shape (12). In *N. punctiforme*, the transition between hormogonia, specialized filaments dedicated to motility, and non-motile vegetative filaments results in dramatic alterations in cell shape between more spherical and rod-like cell morphology, along with the appearance of tapered cells at the filament termini in hormogonia (Fig. S1) (13). In both cases, evidence suggests that the transition to rod morphology is accompanied by transcriptional upregulation of *mreB*, indicating that this morphological plasticity is driven at least partly by changes in known morphogens (14, 15). However, it is also possible that this morphological plasticity involves novel morphogens that have not yet been characterized. For instance, a recent study identified a putative cell-wall hydrolase conserved specifically in filamentous cyanobacteria that is essential for the development of tapered cells at the filament termini in hormogonia of *N. punctiforme* (16).

In this study, we identify and characterize FcmB, a new family of BALPs that, like MreB, is essential for rod morphology in the filamentous cyanobacterium *N. punctiforme*. While functionally similar to MreB, FcmB is evolutionarily distinct and appears to have arisen via horizontal gene transfer of a ParM-type BALP. FcmB is highly conserved in filamentous cyanobacteria but is absent in unicellular strains, indicating that the acquisition of FcmB may be a critical factor facilitating the evolution of morphological plasticity in filamentous cyanobacteria.

## RESULTS

### FcmC indirectly affects motility

While performing an ongoing transposon mutagenesis screen to identify genes involved in hormogonium development and motility (17), we identified two mutants with transposon insertions in Npun_R4472 and Npun_R4471. Based on the results presented below, we have designated these locus tags with the gene names *fcmB* (filamentous cyanobacteria mreB-like) and *fcmC,* respectively. To confirm that these genes play a role in motility, unmarked, in-frame deletion strains were created for each. The Δ*fcmB* strain did not display a reduction in motility in colony spreading assays, but the colonies were qualitatively distinct from the wild type (Fig. S2A and B). In contrast, the Δ*fcmC* strain displayed a severe reduction in motility with very minimal migration away from the inoculation site (Fig. S2A and B). Colony spreading of the Δ*fcmC* strain was partially restored by the introduction of a shuttle vector expressing *fcmC* from the *petE* promoter (Fig. S2A and B). This is consistent with previous observations that complementation of *N. punctiforme* motility-deficient mutants in this manner does not typically completely restore motility to wild-type levels (15) and verifies that deletion of *fcmC* was responsible for the observed phenotype. However, time-lapse microscopy of hormogonia induced from liquid cultures revealed that the Δ*fcmC* strain retained substantial motility at the level of individual filaments, with the primary distinction between wild-type and Δ*fcmC* hormogonia being that wild-type filaments tend to be straighter and travel on a linear trajectory, whereas the Δ*fcmC* hormogonia were often bent and moved in arc-like or circular trajectories (Fig. S2C and D; Movie S1). Collectively, these findings indicate that neither *fcmB* nor *fcmC* plays a key role in the physiology of motility but that *fcmC* indirectly reduces motility by diminishing the total displacement of motile hormogonia.

### FcmB is a BALP related to plasmid partitioning systems

**Fig 1:**
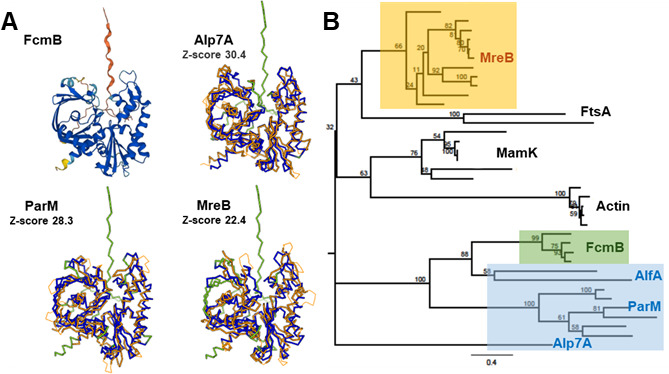
The FcmB family of BALPs. (**A**) The predicted structure of FcmB from the AlphaFold database and structural alignments between FcmB and the BALPs Alp7A (5EC0), ParM (6IZR), and MreB (4CZM) were generated using DALI. For the AlphaFold structure, per-residue model confidence score (pLDDT) >90 = dark blue, >70 = light blue, >50 = yellow,and <50 = orange. For structural alignments, the structure of the indicated protein is depicted in blue, regions of FcmB that closely align with the indicated protein are depicted in orange, and regions that do not align are depicted in green. Z-scores for the structural alignments are indicated. (**B**) Molecular phylogeny of FcmB in relation to other BALP families. The MreB family is highlighted in orange, the plasmid partitioning family in blue, and the FcmB family in green. Protein alignment was performed using the Muscle (22) plugin available in Geneious Prime (ver. 2023.2.1). The phylogenetic tree was generated using the RAxML (23) plugin with the following options: -m PROTGAMMABLOSUM62 -f a -x 1 N 100 p 1, comparing the sequences of FcmB from *N. punctiforme* and three other filamentous cyanobacteria to 32 BALPs from various families (as indicated). For a full list of BALPs used in the comparison, see Table S3. For % identity and % positive matrices of pairwise comparisons among these BALPs, see Data Set S1.

The *fcmB* gene is encoded on the *N. punctiforme* chromosome, and FcmB is annotated in the img.jgi database as a ParM plasmid segregation actin-type ATPase (18). A BLAST search indicated that it contains an ASKHA_NBD_ParM-like domain (cd10227) with high conservation of the nucleotide-binding site residues (Fig. S4) (19). The *fcmC* gene is encoded immediately downstream of *fcmB* in the same orientation, and FcmC is annotated as a ParR plasmid segregation centromere-binding protein (18), although a BLAST search did not identify any conserved domains, and img.jgi does not provide any conserved domains or protein families associated with FcmC. To further investigate whether FcmB is truly a BALP, we analyzed the predicted structure of FcmB from the AlphaFold database (20). The predicted structure was of high confidence, except for the very N- and C-terminal regions and appeared similar to other known BALPs (Fig. 1A). A DALI search (21) using the predicted FcmB structure returned several known BALPs with high Z-scores, including ALP7A, ParM, and MreB, further supporting the hypothesis that FcmB is a BALP (Fig. 1A). While MamK was not returned in the DALI search, targeted alignment of FcmB and MamK also indicated structural similarity, although lower than observed for the other BALPs (Fig. S3). Molecular phylogeny of FcmB from *N. punctiforme* and FcmB orthologs from three additional filamentous cyanobacteria indicates that FcmB forms a distinct clade that evolved from plasmid partitioning type BALPs and is most closely related to the AlfA family (Fig. 1B).

To determine the conservation of FcmB and FcmC in the cyanobacterial phylum, we investigated the presence of orthologs encoded in the genome of 126 cyanobacterial species used in the comprehensive study of cyanobacterial phylogeny by Shi et al. (24). The results indicate that FcmB is highly conserved in many filamentous cyanobacteria, including all of the developmentally complex heterocyst-forming species, but is completely absent in unicellular strains (Fig. S5A). FcmC shows a highly similar conservation pattern, being present in most species that encode FcmB and absent in all species that lack FcmB (Fig. S5A). A small number of species encode FcmB but not FcmC, although it is possible that low sequence identity between the FcmC orthologs accounts for this absence. We manually investigated the *fcmBC* locus from several filamentous cyanobacteria and found that in all cases the genes were encoded in the chromosome, rather than on a plasmid, and maintained the same *fcmB-fcmC* orientation (Fig. S5B). Collectively, these results imply that FcmB is a BALP that evolved from the plasmid partitioning clade, and that *fcmB* and *fcmC* were acquired via horizontal gene transfer and subsequently integrated into the chromosome of filamentous cyanobacteria.

### *fcmB* and *fcmC* are required for rod morphology

**Fig 2:**
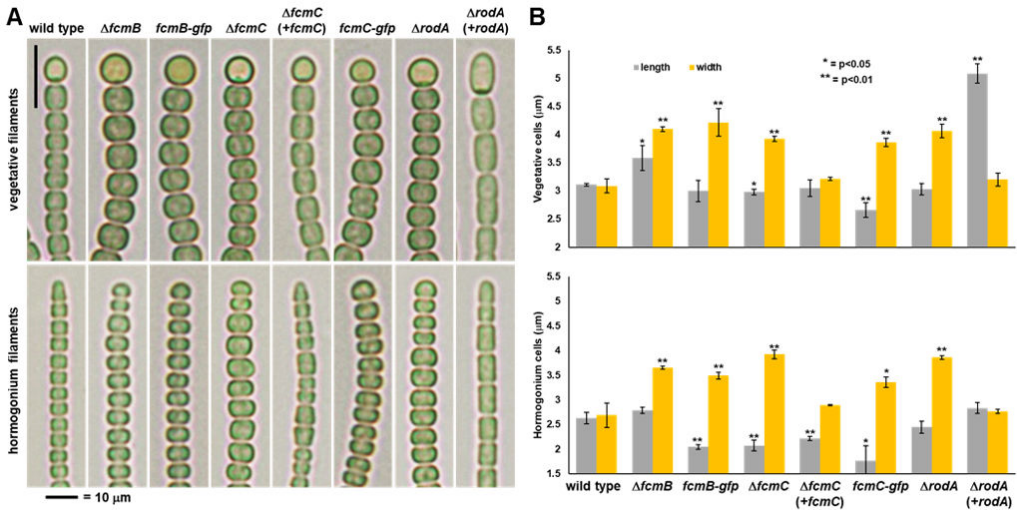
Cell morphology of the Δ*fcmB* and Δ*fcmC* strains. (**A**) Light micrographs of vegetative and hormogonium filaments for strains as indicated. For all vegetative filaments depicted, the terminal cell is a heterocyst. (**B**) Quantification of cell length and width for vegetative and hormogonium filaments for strains as indicated. *P*-values based on Student’s *t*-test for pairwise comparisons of each strain to the wild type.

As described above, deletion of *fcmC* has an indirect effect on motility. To further explore this, we examined the morphology of both vegetative and hormogonium filaments in the Δ*fcmB* and Δ*fcmC* strains (Fig. 2A and B). Compared to the wild type, cells of both vegetative filaments and hormogonia were dramatically wider and rounder in appearance, and hormogonia no longer exhibited tapered terminal cells. This phenotype is similar to that reported for mutations that disrupt the rod complex (9–11). For comparison, we generated a strain with an unmarked, in-frame deletion of the *rodA* gene (Npun_F5165). To our knowledge, *rodA* mutants have not been studied in cyanobacteria, but would be expected to disrupt rod-complex function in a similar manner to other rod-complex genes, such as *mreB*. The cell morphology of the Δ*rodA* strain was highly similar to that of the Δ*fcmB* and Δ*fcmC* mutants. Both the Δ*fcmC* and Δ*rodA* mutants could be complemented by reintroduction of the corresponding gene on a shuttle vector, although complementation of Δ*rodA* also resulted in substantial cell elongation (Fig. 2A and B). Attempts to complement the Δ*fcmB* mutant were unsuccessful due to technical challenges. Introduction of a shuttle vector expressing *fcmB* from the *petE* promoter into the Δ*fcmB* strain failed to produce viable colonies, while attempts to construct a shuttle vector expressing *fcmB* from its native promoter were unsuccessful due to apparent toxicity in *E. coli*. However, reintroduction of *fcmC* into the Δ*fcmB* strain failed to restore wild-type morphology, indicating that the phenotype of the Δ*fcmB* strain cannot be attributed solely to any polar effects on the expression of *fcmC* (Fig. S6). DAPI staining was also employed to determine whether FcmB or FcmC affects chromosome segregation, given that they likely evolved from a plasmid partitioning system, but no obvious alteration in nucleoid distribution was observed in the mutants (Fig. S7). Strains with the chromosomal allele of *fcmB* or *fcmC* replaced with alleles encoding C-terminal GFP fusions were also constructed, but in both cases, the fusion proteins appear non-functional, as the cell morphology more closely resembles that of the mutant strains than the wild type (Fig. S2A and B).

Current understanding of rod-complex function posits that MreB forms membrane-associated filaments which, in turn, spatially constrain cell wall synthesis along the length of the cell to produce rod morphology (6). To determine whether FcmB and FcmC might function in a similar way, fluorescent vancomycin (Van-FL) labeling (25) was employed to visualize the sites of active cell wall synthesis in the mutant strains. Van-FL labeling attempts with vegetative filaments were unsuccessful due to very low labeling even after 24 h of incubation. However, Van-FL labeling could be visualized in developing hormogonia, likely due to the extensive cell wall remodeling that occurs during this transition. Because the overall labeling intensity was highly variable between filaments, we quantified the fluorescence at both the septum and along the length of cells and calculated the length/septum ratio for each cell measured for normalization. The results indicate that both Δ*fcmB* and Δ*fcmC* have reduced cell wall synthesis along the length of the cell (Fig. S8). The Δ*rodA* strain also displayed a reduction in cell wall synthesis along the length of cells, although this was not found to be statistically significant (Fig. S8). Collectively, these results indicate that *fcmB* and *fcmC* are essential for rod morphology in *N. punctiforme* and that they directly or indirectly influence cell wall synthesis along the length of the cell.

In wild-type *N. punctiforme*, rod morphology is more prominent in hormogonia than in vegetative filaments, and known rod complex components are transcriptionally upregulated in developing hormogonia (15). To determine whether transcription of *fcmB* and *fcmC* is correlated with enhanced rod morphology, we examined the transcriptional profile of these genes in developing hormogonia using previously published RNAseq (15) and CAPseq (26) data sets (Fig. S9A and B). Both *fcmB* and *fcmC* were found to be transiently upregulated in developing hormogonia, with expression peaking at 6 h post-induction. Enhanced expression of these genes was not observed when either of the hormogonium-specific sigma factors *sigJ* or *sigC* was deleted. Given that hormogonium development is driven by a hierarchical sigma factor cascade with *sigC* expression dependent on *sigJ* (15), these results imply that upregulation of *fcmB* and *fcmC* in developing hormogonia is most directly dependent on *sigC*. Read coverage from RNAseq and transcriptional start site (TSS) mapping from CAPseq indicates that *fcmB* and *fcmC* are transcribed as an operon from a pair of TSS upstream of *fcmB* (Fig. S9B). Read coverage for both TSSs is reduced in the Δ*sigJ* and Δ*sigC* strains, again consistent with the theory that transcription of the operon is most directly dependent on SigC.

### Localization of FcmB and FcmC

**Fig 3:**
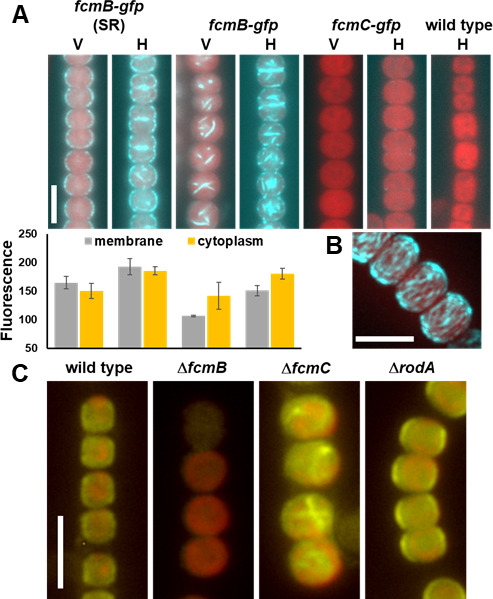
Localization of FcmBC. (**A**) Fluorescence micrographs of FcmB-GFP or FcmC-GFP (cyan) and autofluorescence (red) in vegetative (V) or hormogonium (H) filaments for strains as indicated. Membrane and cytoplasmic fluorescence (A.U.) was quantified for FcmB-GFP and depicted beneath each corresponding strain and filament type. White bar = 5 µm. (**B**) Maximal intensity projection of FcmB-GFP (cyan) and autofluorescence (red) from SIM. White bar = 5 µm. (**C**) Fluorescence micrographs depicting localization of FcmB by immunofluorescence (yellow) and autofluorescence (red) in strains as indicated. White bar = 5 µm.

Although the *fcmB-gfp* and *fcmC-gfp* strains described above did not produce functional fusion proteins, based on the failure to produce wild-type cell morphology, immunoblot analysis with α-GFP antibodies confirmed that these strains expressed full-length fusion proteins (Fig. S10). Thus, we attempted to use them to investigate the possible localization of FcmB and FcmC. Additionally, we analyzed an intermediate, single recombinant strain, *fcmB-gfp* (SR), generated during the construction of *fcmB-gfp*, which contains both the wild-type and *fcmB-gfp* alleles. In vegetative filaments of the *fcmB-gfp* (SR) strain, FcmB-GFP localized to discrete foci associated with the cell membrane and was typically absent from the septa (Fig. 3A). In hormogonia, a similar localization pattern was observed, with some accumulation of FcmB-GFP in the cytoplasm and occasional septal localization (Fig. 3A). An overall increase in fluorescent intensity in hormogonia compared to vegetative filaments was also observed, consistent with transcriptional data showing upregulation of *fcmB* in hormogonia (Fig. 3A). We postulate that the appearance of FcmB-GFP at the septa is a result of cell division over mid-cell regions previously occupied by FcmB-GFP, given that developing hormogonia undergo rapid, synchronous cell division. In contrast, the *fcmB-gfp* strain, which only expresses the *gfp*-fusion allele, produced what appeared to be filaments of FcmB-GFP that accumulated within the cytoplasm (Fig. 3A). Overall accumulation of FcmB-GFP was also higher in hormogonia for this strain (Fig. 3A). Given the dramatic change in FcmB-GFP localization in the presence, versus absence of wild-type FcmB, it is likely that the membrane localization observed in the presence of wild-type FcmB represents the proper localization in the cell and is a result of heterocomplexes between wild-type FcmB and FcmB-GFP. To further investigate this localization pattern, three-dimensional imaging with structured illumination microscopy (SIM) was employed. The results indicate that in the *fcmB-gfp* (SR) strain, FcmB localizes to filaments that wrap around the cell, mostly perpendicular to the long axis of the filament (Fig. 3B; Fig. S11).

In contrast with FcmB-GFP, fluorescent signal from FcmC-GFP was undetectable in vegetative filaments, but in hormogonium filaments, dim fluorescent foci that appeared to be associated with the cell membrane could be observed (Fig. 3A). The low levels of fluorescence derived from FcmC-GFP are consistent with the results from immunoblot analysis, indicating a much lower abundance for FcmC-GFP compared to FcmB-GFP (Fig. S10).

To provide further evidence on the proper localization of FcmB, we raised antibodies to visualize FcmB localization by immunofluorescence. Immunoblot analysis confirmed that these antibodies were highly specific for FcmB and that FcmB levels are higher in hormogonia compared to vegetative filaments (Fig. S12). Two distinct isoforms of FcmB with slightly different molecular weights were also observed, possibly indicating post-translational modification or processing. Immunofluorescence detection of FcmB in the wild type displayed a similar localization pattern to that of the *fcmB-gfp* (SR) strain, confirming that FcmB is in fact localized to the membrane (Fig. 3C). We subsequently employed immunofluorescence to determine whether deletion of *fcmC* or *rodA* affected FcmB localization. In the Δ*fcmC* strain, FcmB localized to filaments in the cytoplasm (Fig. 3C), reminiscent of those visualized in the *fcmB-gfp* strain, indicating that *fcmC* is essential for proper localization of FcmB. In contrast, deletion of *rodA* had no apparent effect on FcmB localization (Fig. 3C). These results imply that FcmB forms membrane-associated filaments that wrap around the circumference of the cell perpendicular to the long axis of the trichome, and that the proper localization of these FcmB filaments is dependent on FcmC.

While investigating the localization of FcmB-GFP in the *fcmB-gfp* (SR) strain, we also noticed an intriguing phenomenon. Prolonged exposure to the excitation light used for fluorescent imaging (i.e*.*, 10-s exposure) triggered delocalization of FcmB-GFP from the membrane and subsequent diffuse accumulation in the cytoplasm (Fig. S13). Notably, this phenomenon was only observed in vegetative cells, but is not heterocysts, suggesting the phenomenon is dependent on the light-harvesting photosynthetic complexes, which are largely absent in heterocysts (27). Additionally, we tested the effect of the proton ionophore carbonyl cyanide m-chlorophenyl hydrazone (CCCP) on FcmB-GFP in this strain to determine whether membrane polarization might influence localization and found a similar effect to that of light exposure (Fig. S13). In the case of CCCP treatment, it is possible that this is an indirect effect due to ATP depletion. However, exposure to high-intensity light would not be expected to deplete ATP levels, and previous work has posited that light-driven changes in membrane polarity might function as a signal for other processes such as motility (28). While we cannot draw any firm conclusions on the underlying physiology driving this phenomenon, or exclude the possibility that both light and CCCP treatment are inducing more general cellular damage responsible for altering FcmB localization, given the influence of fluorescence imaging on FcmB localization, we did not attempt to pursue time course experiments that would expose the same cells to fluorescence imaging over time to determine whether FcmB localization is dynamic.

### Direct interaction of FcmB and FcmC

**Fig 4:**
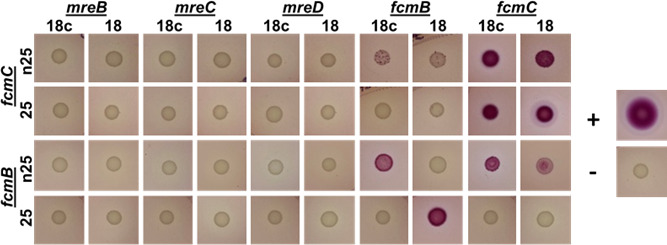
Protein-protein interaction between FcmB, FcmC, and Mre system proteins. BACTH assays on MacConkey medium for BTH101 co-transformed with pUT18 (18) or pUT18c (18 c) and pKT25 (25) or pKNT25 (n25) containing the indicated gene. The positive control (+) contains pUT18-Zip and pKT-Zip, while the negative control (−) contains empty vectors.

Based on the data presented above, as well as current knowledge on ParM-type plasmid partitioning systems (3), it is likely that FcmB forms filaments and that FcmC interacts with the ends of these filaments. Moreover, given that *fcmB* and *fcmC* are essential for rod morphology, it is possible that they directly interact with other rod-complex components. To test these hypotheses, the bacterial adenylate cyclase two-hybrid (BACTH) assay (29) was employed to test for direct interaction between FcmB and FcmC, as well as either FcmB or FcmC with the rod complex components MreB, MreC, and MreD. The results indicate that FcmB and FcmC directly interact, but that neither interacts with MreB, MreC, or MreD (Fig. 4). Importantly, the failure to detect an interaction between FcmB and MreB implies that these two BALPs do not form heterofilaments. Self-interactions were also observed for both FcmB and FcmC, indicating that these proteins form homomultimers. This is consistent with the expectation that FcmB polymerizes to form filaments, and previous work indicating that ParR proteins form multimers (3).

## DISCUSSION

The evidence presented in this study supports the theory that FcmB is a new family of BALPs, which, along with FcmC, is required for rod morphology in *N. punctiforme* and likely other filamentous cyanobacteria. Although the phenotype of the *fcmB* and *fcmC* mutants resembles those reported for *mreB* and other rod-complex genes, *fcmB* does not appear to have evolved via a gene duplication of *mreB*, but rather via horizontal gene transfer of a plasmid partitioning system. This system was then integrated into the genome and evolved a distinct function in cell morphology rather than DNA segregation. Acquisition of this system presumably occurred in a predecessor to the developmentally complex heterocyst-forming cyanobacteria, given that orthologs of FcmBC are found in all heterocyst-forming species as well as a subset of closely related filamentous strains that lack heterocysts.

**Fig 5:**
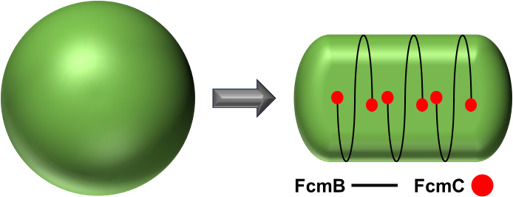
A working model of the FcmBC system. FcmB forms membrane-associated filaments that wrap around the circumference of the cells. FcmC associates with the ends of the FcmB filaments and is essential for proper membrane association of FcmB. The system then promotes the transition from spherical to rod-shaped cells, possibly via spatial regulation of cell wall synthesis or the generation of constrictive force.

Given the data presented, as well as current knowledge of the closely related plasmid partitioning systems, we propose a model (Fig. 5) where FcmB forms membrane-associated filaments that wrap around the circumference of the cell and are capped at the ends by FcmC, and that this complex then promotes rod morphology. The fact that deletion of *fcmC* produces cytoplasmic, rather than membrane-associated filaments of FcmB, implies that FcmC plays a critical role in the proper localization of FcmB to the membrane. This could also explain the mis-localization of FcmB-GFP in the absence of untagged FcmB, as the C-terminal GFP tag may interfere with FcmB-FcmC interaction. However, the mechanism by which this system promotes rod morphology is not clear. One possibility is that the FcmBC system functions in a similar manner to MreB, coordinating the site of cell wall synthesis. Van-FL labeling of lateral peptidoglycan synthesis, which was reduced in the *fcmB* and *fcmC* mutants, is consistent with this hypothesis. But this could be an indirect effect, and the absence of detectable protein-protein interactions between FcmB or FcmC and MreB, MreC, or MreD by BACTH, while not conclusive, does not support direct interaction between the FcmBC system and the rod complex. Notably, while the localization of MreB has not been determined in *N. punctiforme*, it has been examined in the closely related *Nostoc* sp. PCC 7120, where it was found to primarily accumulate at the cell poles (11), suggesting the systems are restricted to different parts of the cell.

An intriguing alternative hypothesis is that the FcmBC system may exert constrictive forces on the cell, essentially functioning as a girdle to cinch the cell into a rod shape. This hypothesis is particularly relevant when considering that developing hormogonia undergo rapid reduction of the cell width, as it is difficult to conceive how this could be achieved strictly based on spatial regulation of cell wall synthesis and degradation, which could elongate the cell, but would not lead to constriction. The constriction hypothesis would also help explain why two distinct families of BALPs are required for rod morphology. While some bacterial species, such as *B. subtilis*, contain multiple paralogs of *mreB*, these typically show some functional redundancy (30). In contrast, based on the results of this study, as well as others on the role of *mreB* in the closely related filamentous cyanobacterium *Nostoc* sp. PCC 7120 (10, 11), it appears that both *mreB* and *fcmB* are essential to generate rod-shaped cells, which is consistent with the idea that they play distinct roles influencing cell morphology. While more work is required to determine how the FcmBC system influences cell morphology, the results presented indicate that this system is critical for the phenotypic plasticity observed in filamentous cyanobacteria. Notably, the filamentous cyanobacteria that lack the FcmBC system are in subsection III, which does not typically display the phenotypic plasticity observed in the more developmentally complex subsections IV and V species (31), all of which encode FcmBC orthologs. The findings from this study may also help to explain why some cyanobacteria that encode rod-complex proteins, such as the unicellular cyanobacterium *Synechocystis* sp. PCC 6803, fail to display rod morphology, as they lack the FcmBC system. However, it should be noted that there are examples of rod-shaped cyanobacteria, such as *Synechococcus elongatus* sp. PCC 7942, which encodes *mreB*, but not *fcmB*. Thus, the FcmBC system is not strictly required for rod morphology in all cyanobacteria.

## MATERIALS AND METHODS

### Strains and culture conditions

For a detailed description of the strains used in this study, refer to Table S2. *N. punctiforme* ATCC 29133 and its derivatives were cultured in Allen and Arnon medium diluted fourfold (AA/4), without the supplementation of fixed nitrogen, as previously described (32), with the exception that 4 and 10 mM sucralose were added to liquid and solid medium, respectively, to inhibit hormogonium formation (33). For hormogonium induction for phenotypic analysis, the equivalent of 30 µg chlorophyll *a* (Chl *a*) of cell material from cultures at a Chl *a* concentration of 10–20 µg ml^−1^ was harvested at 2,000 × *g* for 3 min, washed two times with AA/4, and resuspended in 2 mL of fresh AA/4 without sucralose. For selective growth, the medium was supplemented with 50 µg mL^−1^ neomycin. *E. coli* cultures were grown in lysogeny broth (LB) for liquid cultures or LB supplemented with 1.5% (wt/vol) agar for plates. Selective growth medium was supplemented with 50 µg mL^−1^ kanamycin, 50 µg mL^−1^ ampicillin, and 15 µg mL^−1^ chloramphenicol.

### Plasmid and strain construction

For a detailed description of the plasmids, strains, and oligonucleotides used in this study, refer to Table S2. All constructs were sequenced to ensure fidelity.

To construct plasmids for in-frame deletions, approximately 900 bp of flanking DNA on either side of the gene and several codons at the beginning and end of the gene were amplified via overlap extension PCR (see Table S2 for details of plasmids and primers) and cloned into pRL278 (34) as BamHI-SacI fragments using restriction sites introduced on the primers.

To construct plasmid pGAP112 for the replacement of the chromosomal allele of *fcmB* with a C-terminal *gfpuv*-tagged variant, the coding region of *fcmB* was amplified via PCR and cloned into pSCR569 (35), as a BamHI-SmaI fragment using restriction sites introduced on the primers. Approximately 900 bp of DNA downstream of the *fcmB* stop codon was then amplified via PCR and cloned into this plasmid as a SpeI-SacI fragment using restriction sites introduced on the primers. To construct plasmid pGAP111 for replacement of the chromosomal allele of *fcmC* with a C-terminal *gfpuv*-tagged variant, the coding region of *fcmC* and approximately 900 bp upstream of the start codon were amplified via PCR and cloned into pSCR569 (35), as a BamHI-SmaI fragment using restriction sites introduced on the primers. Approximately 900 bp of DNA downstream of the *fcmC* stop codon was then amplified via PCR and cloned into this plasmid as a SpeI-SacI fragment using restriction sites introduced on the primers.

To construct a mobilizable shuttle vector containing *fcmC* or *rodA* expressed from the *petE* promoter, the coding regions were amplified via PCR and cloned into pDDR155 (36) as BamHI-SacI fragments, replacing the *hmpA-gfp* coding region, using restriction sites introduced on the primers.

To construct plasmids encoding proteins of interest fused to either the T18 or T25 fragment of *Bordetella pertussis* adenylate cyclase for BACTH analysis (29, 37), the coding region of each gene was amplified via PCR and cloned into either pUT18/pUT18c or pKT25/pKNT25 as BamHI-KpnI fragments using restriction sites introduced on the primers.

Gene deletion was performed as previously described (38) with *N. punctiforme* cultures supplemented with 4 mM sucralose to inhibit hormogonium development and enhance conjugation efficiency (17, 33). To construct UOP177, UOP180, and UCCS100, plasmids pDDR470, pDDR477, and pDDR478, respectively, were introduced into wild-type *N. punctiforme* ATCC29133. To construct UCCS116, plasmid pGAP11 was introduced into UOP180. To construct UCCS114 and UCCS125, plasmid pGAP112 was introduced into wild-type *N. punctiforme* ATCC29133.

### BACTH assays

The BACTH assay (29, 37) was employed to probe protein-protein interaction between various proteins. BTH101 (adenylate cyclase-deficient) *E. coli* strains transformed with appropriate plasmids were streaked onto LB agar plates containing 100 µg/mL ampicillin and 50 µg/mL kanamycin and incubated at 30°C for 24 h. Qualitative assays on MacConkey agar were performed as previously described (39), with several modifications as described (40).

### Motility assays

Plate and time-lapse motility assays were performed as previously described (41). Briefly, for plate motility assays, colonies were transferred from AA/4 solid medium (1% noble agar) containing 5% sucrose, to suppress hormogonium development, to the surface of AA/4 solid medium (0.5% noble agar) without sucrose. Plates were incubated for 2 days under light. For time-lapse motility assays, following standard hormogonium induction from liquid cultures, 2 µL of culture was spotted onto the surface of AA/4 solid medium (0.5% noble agar), overlayed with a cover slip, and imaged at 15-s intervals. Both plate and time-lapse motility assays were imaged with a Leica SD9 dissecting microscope equipped with a Leica Flexcam C3 camera controlled by Leica LAS X software.

### Microscopy

Light and fluorescence microscopy was performed with an EVOS M5000 fluorescence microscope (Life Technologies) equipped with a 40× or 63× objective lens. Excitation and emission were as follows: EVOS light cube, Nrw 405 (AMEP4857: excitation 390/18 nm, emission 525/50 nm) for GFPuv; EVOS light cube, GFP (AMEP4651: excitation 470/22 nm, emission 525/50 nm) for Van-FL and α-rabbit fluorescein secondary antibodies; DAPI (AMEP4650: excitation 357 ± 44 nm, emission 447/60 nm) for DAPI stained nucleoids; and EVOS Light Cube, RFP (AMEP4652: excitation 531/40 nm, emission 593/40 nm) for cellular autofluorescence. DAPI was added to the media at 1 µg/µL to visualize nucleoids. For CCCP treatment, 100 µL of culture was incubated for 15 min in AA/4 containing 10 µM CCCP, 0.1% DMSO, or AA/4 with 0.1% DMSO as a control, centrifuged at 2,000 × *g*, the supernatant discarded, and the cells immediately fixed in 100 µL of 4% paraformaldehyde for 2 h. Fixed cells were subsequently centrifuged for 1 min at 16,000 × *g*, the paraformaldehyde removed, and the cells subsequently washed twice with phosphate-buffered saline. The cells were then resuspended in 25 µL EverBright hardset mounting medium, 10 µL was spotted onto a slide, overlayed with a coverslip, and incubated overnight at room temperature in the dark. Quantification of membrane and cytoplasmic GFP fluorescence was performed using ImageJ. For the membrane fluorescence, a line was drawn along the length of the cell periphery, and for cytoplasmic fluorescence, a circle was drawn within the cytoplasm of the cell, and the average intensity was measured. Measurements were performed on a total of 10 cells for each of 3 biological replicates. For SIM, cells were fixed in 4% paraformaldehyde as described above prior to imaging to avoid imaging effects on FcmB-GFP localization. Prepared cells were subsequently imaged on a home-built super-resolution SIM described previously (42, 43). Briefly, SIM patterns are created by a liquid crystal-on-silicon microdisplay and projected into an IX83 microscope equipped with a UPLSAPO 100×/1.4 NA oil immersion objective (Olympus, Tokyo, Japan). We used a solid-state light source at 470 nm (Spectra-X, Lumencor, Beaverton, OR) and a filter set for GFP (Chroma, Bellows Falls, VT). Fluorescence signals were recorded with a Zyla 4.2+ CMOS camera (Andor, Belfast, Northern Ireland). To create a Z-stack, we used a Piezo-Z stage (ASI, Eugene, OR). We acquired 41 Z-slices with a spacing of 250 nm and an exposure time of 1 s. We processed the data using Maximum a *posteriori* probability methods (44) in the SIMToolbox software package (45). Inspection of the fast Fourier transform (the frequency spectrum) indicates that the resolution in the acquired images reached about 150 nm.

### Van-FL labeling

For vancomycin-BODIPY-FL (Van-FL, Invitrogen V34850) labeling, immediately following standard hormogonium induction, 1 µL of Van-FL in DMSO at a concentration of 2 µg/µL was added to 1 mL of culture and incubated for 24 h under illumination. The cultures were subsequently washed two times with 1 mL of AA/4 to remove unincorporated Van-FL and imaged. Quantification of labeling was performed using ImageJ by drawing a line at the septum between cells or along the length of the periphery of each cell and recording the average intensity, then calculating the intensity ratio of the length/septum for each cell. Measurements were performed on 10 cells from each of three biological replicates.

### Immunoblot analysis

Preparation of *N. punctiforme* cell material, protein extraction, and detection of GFPuv by immunoblot analysis were performed as previously described (46). Briefly, total cellular protein was extracted from cell material equivalent to 30 µg Chl*a* following standard protocols (46), the lysate containing extracted proteins was separated on a 4%–12% SDS-PAGE gel, and then transferred to a nitrocellulose membrane. For detection of FcmB, rabbit monospecific antibodies against the peptide KPRGERYYRPKGASKPTNLD were generated (Monospecific Antibody Production Package, Pacific Immunology) and used at a 1:1,000 dilution, followed by a 1:20,000 dilution of an HRP-conjugated anti-rabbit secondary antibody (Chemicon). Chemiluminescence and phycobilisome autofluorescence, which served as a protein loading control, were imaged using an Azure Biosystems c400 imager (Azure Biosystems Inc.), using the Chemiluminescence and Gel UV302 settings, respectively.

### Immunofluorescence of FcmB

For immunofluorescence detection of FcmB, 1 mL of culture was collected and centrifuged at 2,000 × *g* for 3 min. The supernatant was discarded, and the cells were resuspended in 1 mL of 100% ice-cold methanol and incubated at −20°C for 30 min. 20 µL of cell material in methanol was subsequently spotted onto a poly-L-lysine-coated slide (Poly-Prep Slide, Sigma-Aldrich Co.) and allowed to air dry. The slides were washed for 5 min in TBS (0.1 M Tris, 0.15 M NaCl, pH 7.8) at room temperature and then placed in a moisture chamber for all subsequent steps. The slides were subsequently incubated in lysozyme solution (20 μM Tris, 10 mM EDTA, 0.1% Triton X 100, 10 mg/mL lysozyme, pH 8.0) for 30 min at 37°C. Lysozyme solution was subsequently removed, the slides were washed two times with TBS for 5 min at room temperature, and 500 µL of blocking solution (TBS, 0.1% Tween 20, 3% bovine serum albumin) was applied to the slides for 30 min at room temperature. Blocking solution was removed and the slides were subsequently incubated in 500 µL of primary antibody solution (blocking solution containing a 1:100 dilution of α-FcmB rabbit monospecific antibody) overnight at 4°C. The following day, the primary antibody solution was removed, the slides were washed 3 times for 5 min with TBST (TBS, 0.1% Tween 20) at room temperature, and subsequently incubated overnight at 4°C with 500 µL of secondary antibody solution (blocking solution containing a 1:250 dilution of fluorescein isothiocyanate-conjugated goat anti-rabbit secondary antibody; Sigma-Aldrich Co.). The following day, the secondary antibody solution was removed, the slides were washed 3 times for 5 min with TBST at room temperature, and air-dried. 10 µL of EverBright hardset mounting medium (Biotium) was applied to the slides, overlayed with a coverslip, and allowed to harden overnight at room temperature in the dark prior to imaging.

## SUPPLEMENTAL MATERIAL

Data Set S1 msphere.00499-25-s0001.xlsx Percent identity and percent positive matrices for BALPs used to generate the phylogenetic tree in Fig. 1B.

Supplemental Material msphere.00499-25-s0002.pdf Figures S1-S13, Tables ST1-ST3, and Movie S1 and Data Set S1 legends.

Movie S1 msphere.00499-25-s0003.avi Time-lapse microscopy of motile hormogonia in the wild type, *fcmB* mutant, and *fcmC* mutant strains.

ASM does not own the copyrights to Supplemental Material that may be linked to, or accessed through, an article. The authors have granted ASM a non-exclusive, world-wide license to publish the Supplemental Material files. Please contact the corresponding author directly for reuse.
