# Supplementary material for: A new family of bacterial actin-like proteins regulates cell morphology in a filamentous cyanobacterium
Source: mSphere. 2025 Oct 31;10(11):e00499-25. doi: 10.1128/msphere.00499-25 (PMC12645912; doi:10.1128/msphere.00499-25)
Supplement: Supplemental Material — Figures S1-S13, Tables ST1-ST3, and Movie S1 and Data Set S1 legends. [file msphere.00499-25-s0002.pdf]

Supplementary Materials for:

A new family of bacterial actin-like proteins regulates cell morphology in a filamentous cyanobacterium

Alicia Nguyen[1], Garrett M. Jenkins[1], Peyton D. Brones[1], Gabriel A. Parrett[1], Guy M. Hagen[2], Jeremy M. Bono[1], and Douglas D. Risser[1]*

[1] Department of Biology, University of Colorado Colorado Springs, Colorado Springs, CO 80918 USA

[2] BioFrontiers Center, University of Colorado Colorado Springs, Colorado Springs, CO, 80918 USA

*To whom correspondence should be addressed. Email: drisser@uccs.edu
*ORCID ID: 0000-0003-1981-0049

Table ST1. Families of BALPs with known biological functions

Family	Function
ParM	Plasmid Partitioning (includes Alp7A and AlfA)
MamK	Magnetosome positioning
FtsA	Cell division
MreB	Rod morphology

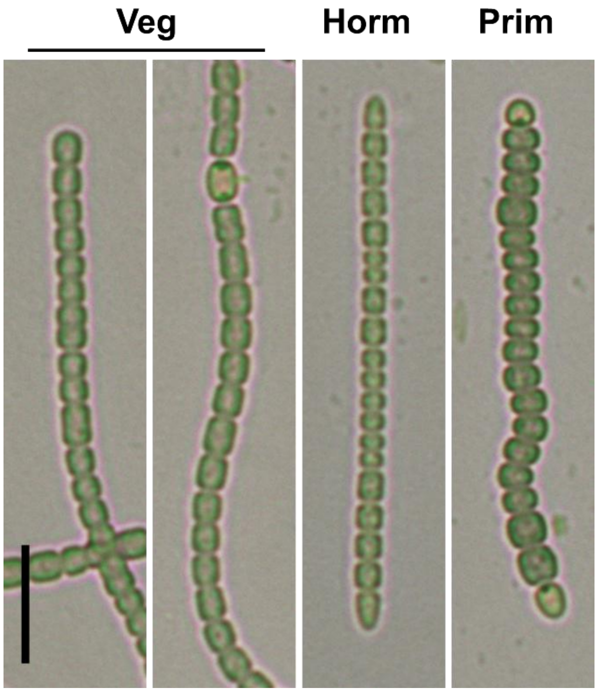

Fig. S1. Cell morphology of various filament types observed in *N. punctiforme*. Vegetative filaments (Veg) are non-motile and contain intercalary heterocysts in the absence of fixed nitrogen. Hormogonia (Horm) differentiate from vegetative filaments and exhibit reduced cell-length and width as well as more rod-shaped morphology and tapered filament termini. Hormogonia subsequently differentiate into primordia (Prim) which cease motility, develop heterocysts at the filament termini and exhibit larger, more coccoid cells. Primordia will subsequently develop into vegetative filaments. Bar = 10 μm.

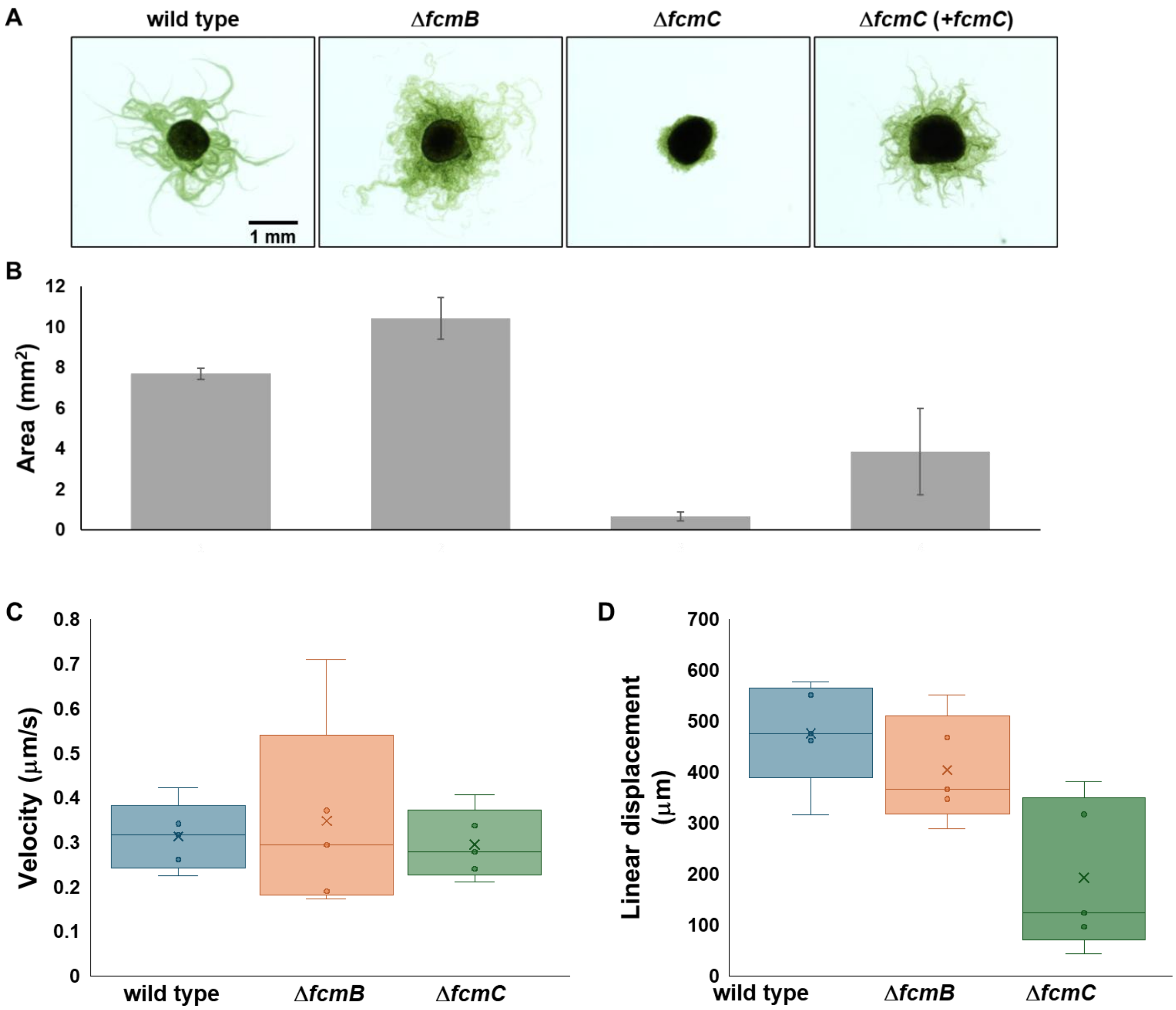

Fig. S2. Motility in the Δ*fcmB* and Δ*fcmC* mutant strains. (**A**) Images of colony spreading assays for strains as indicated. (**B**) Quantification of colony spreading for strains as indicated in A. n = 3, error bars = +/- 1 S.D. (**C**) Average velocity and (**D**) linear displacement for strains as indicated, derived from individual motile filaments depicted in Movie S1. n=5.

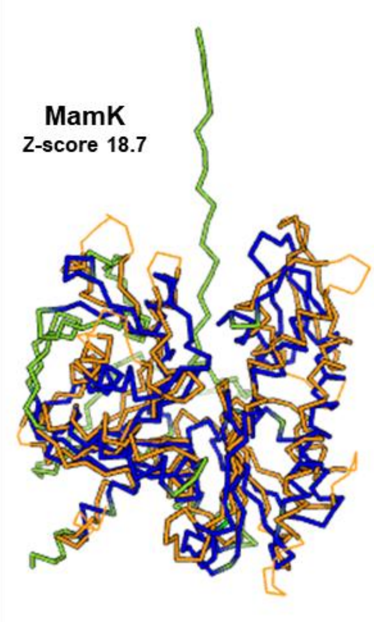

Fig. S3. Structural alignments between FcmB and MamK (5JYG-A) generated using DALI. For the For structural alignment, MamK is depicted in blue, regions of FcmB that closely align with MamK are depicted in orange, and regions that do not align are depicted in green. Z-score for the structural alignment is indicated.

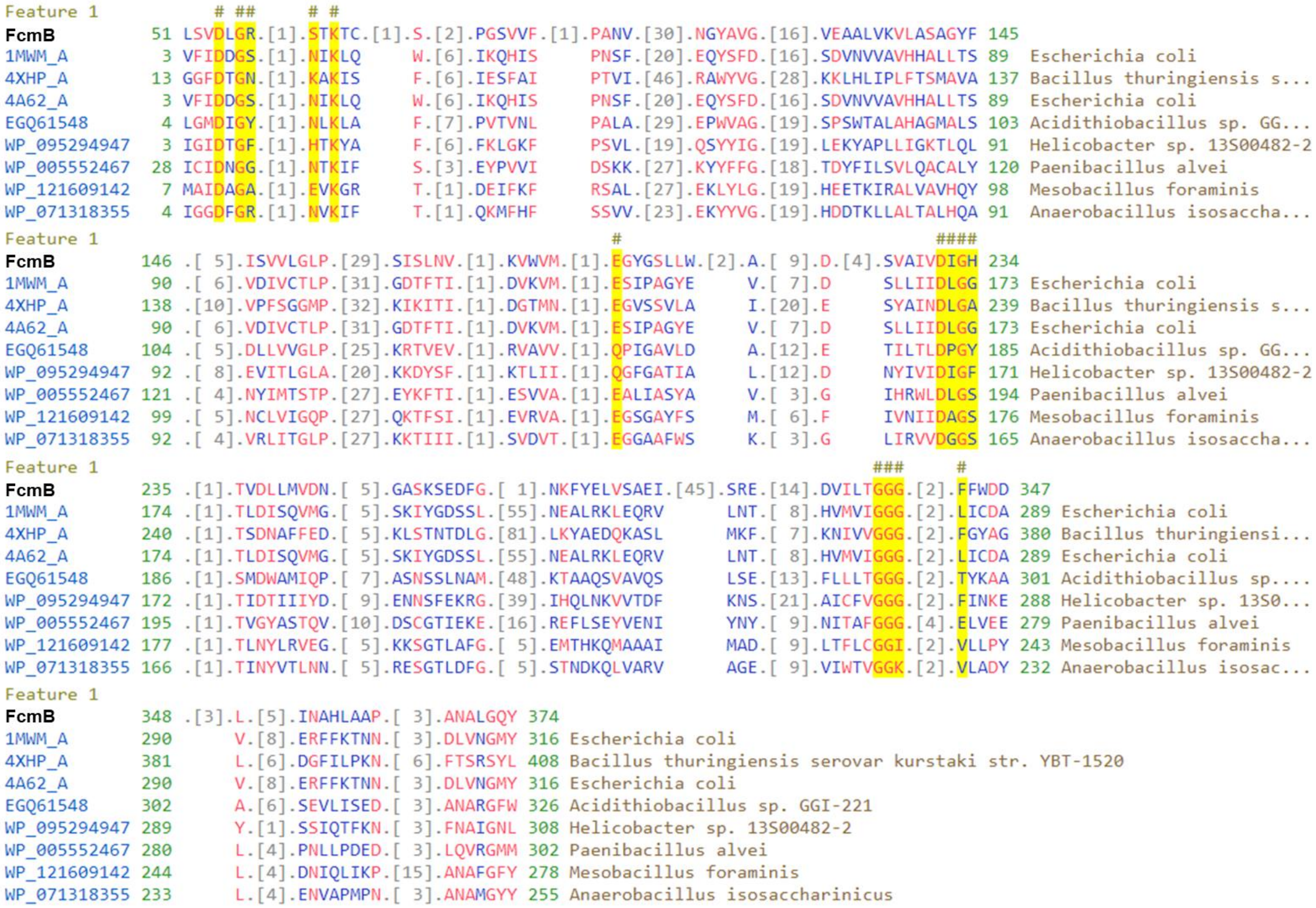

Fig. S4. Conservation of ATP-binding residues in FcmB. Depicted are the results from an alignment of FcmB with other BALPs containing the conserved domain cd10227: ASKHA_NBD_ParM-like. ATP-binding residues are indicated with # and highlighted in yellow. Alignment was generated using the Conserved Domain Database **(1)**.

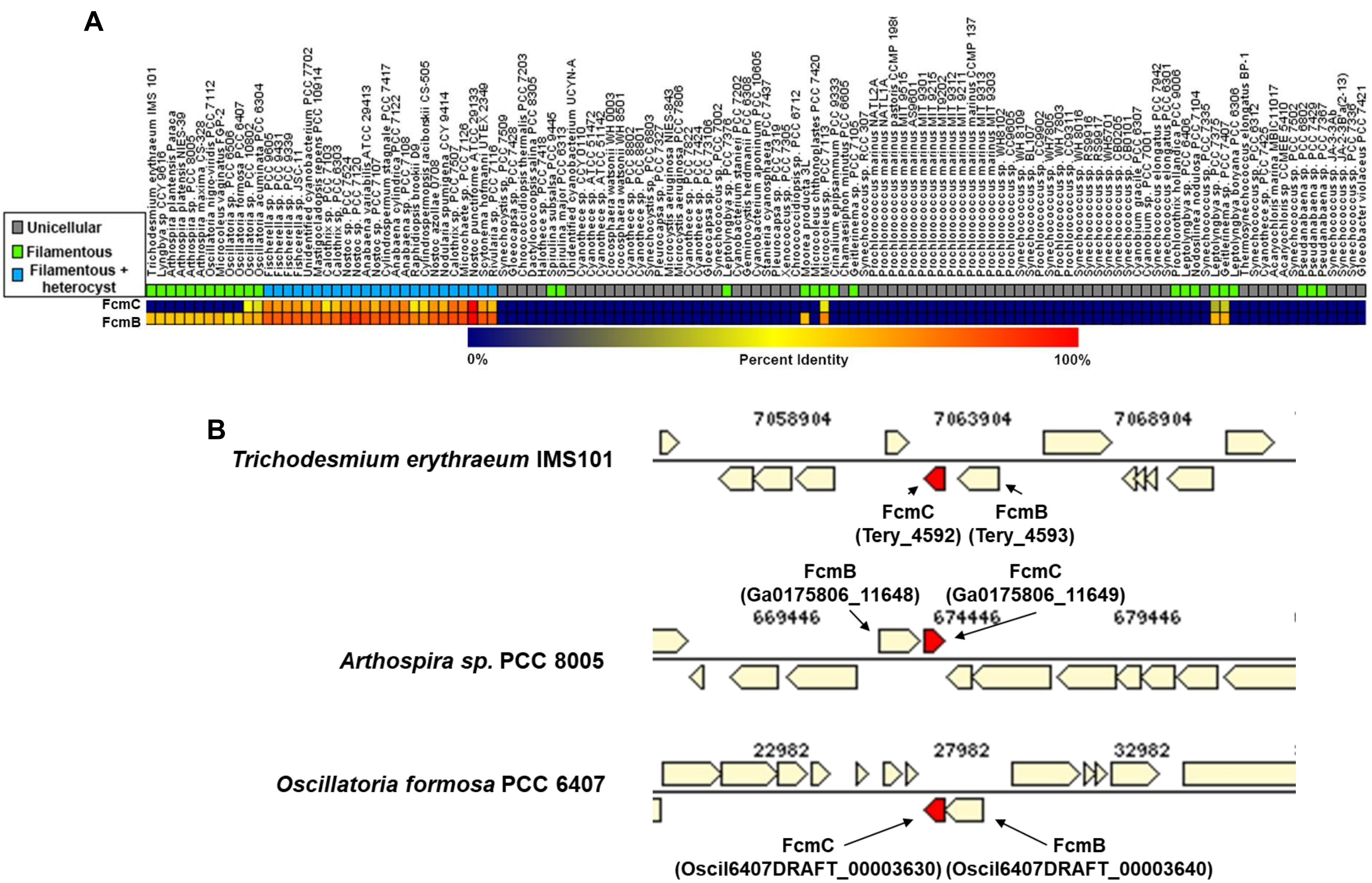

Fig. S5. Conservation of FcmBC in cyanobacteria. (**A**) Heat map depicting the percent identity for orthologs of *N. punctiforme* FcmB and FcmC in cyanobacteria, derived from data reported by Cho *et al*. (2). Species organization based on the phylogeny reported by Shih *et al*. (3). (**B**) Genomic regions of select cyanobacteria where FcmC was identified by manual BLAST searches.

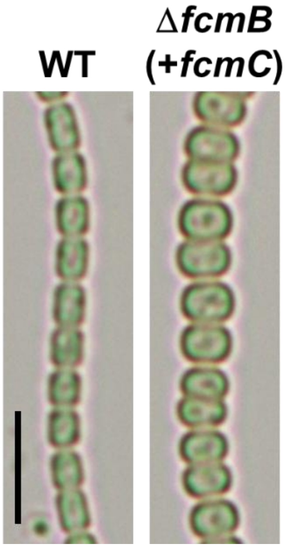

Fig. S6. *fcmC* fails to complement the *fcmB* mutant. Light micrographs of the wild type (WT) and Δ*fcmB* strain containing a plasmid expressing *fcmC* from the *petE* promoter (+*fcmC*). Bar = 10 μm.

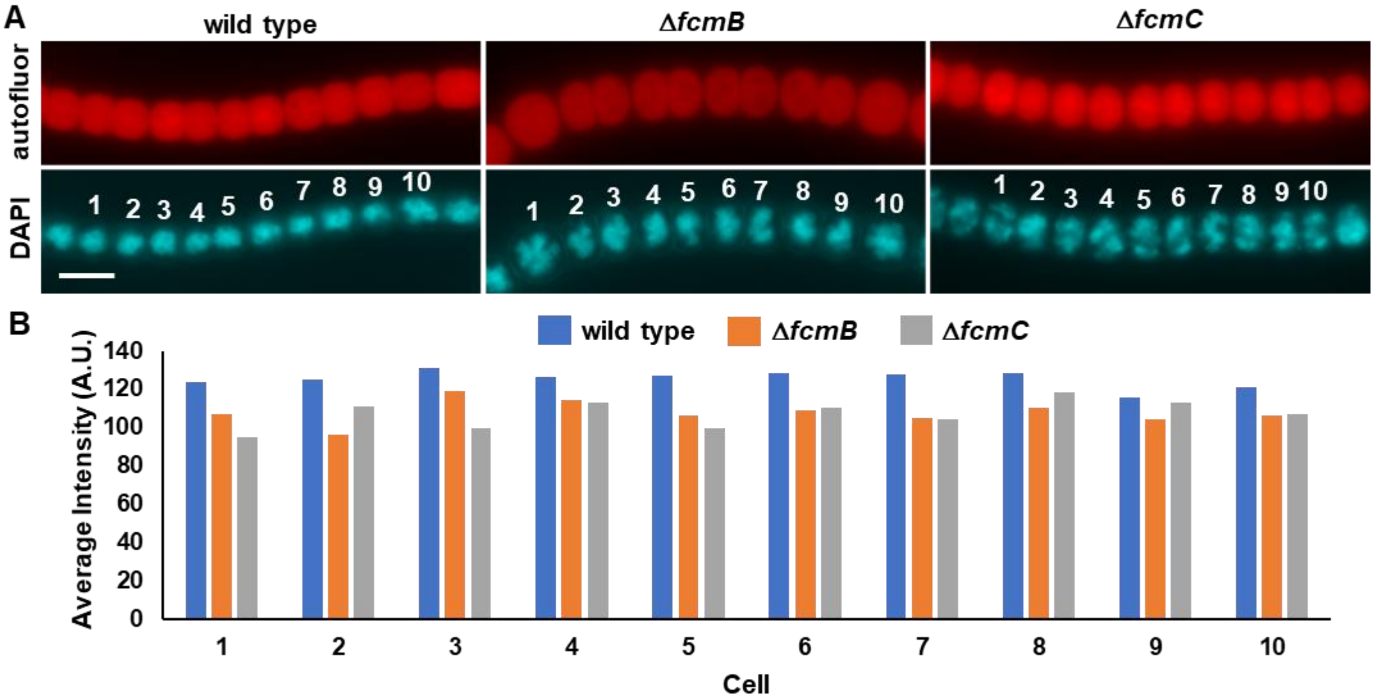

Fig. S7. DAPI staining of nucleoids in the wild type, Δ*fcmB* and Δ*fcmC* strains. (**A**) Fluorescence micrographs of autofluorescence (autofluor) and DAPI stained nucleoids in strains as indicated. White bar = 5 μm. (**B**) Quantification of DAPI fluorescence. The x-axis corresponds to the numbered cells in A.

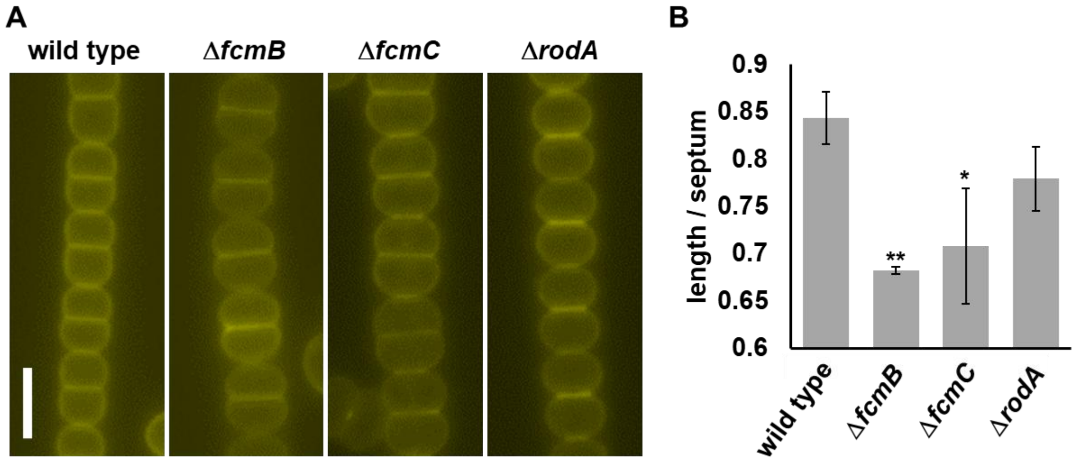

Fig. S8. Van-FL labeling of cell wall synthesis in hormogonia. (**A**) Fluorescence micrographs of strains as indicated. White bar = 5 μm. (**B**) Quantification of Van-FL labeling. * p<0.05, ** p<0.01 based on student's t-test for pairwise comparisons between the wild type and each mutant strain.

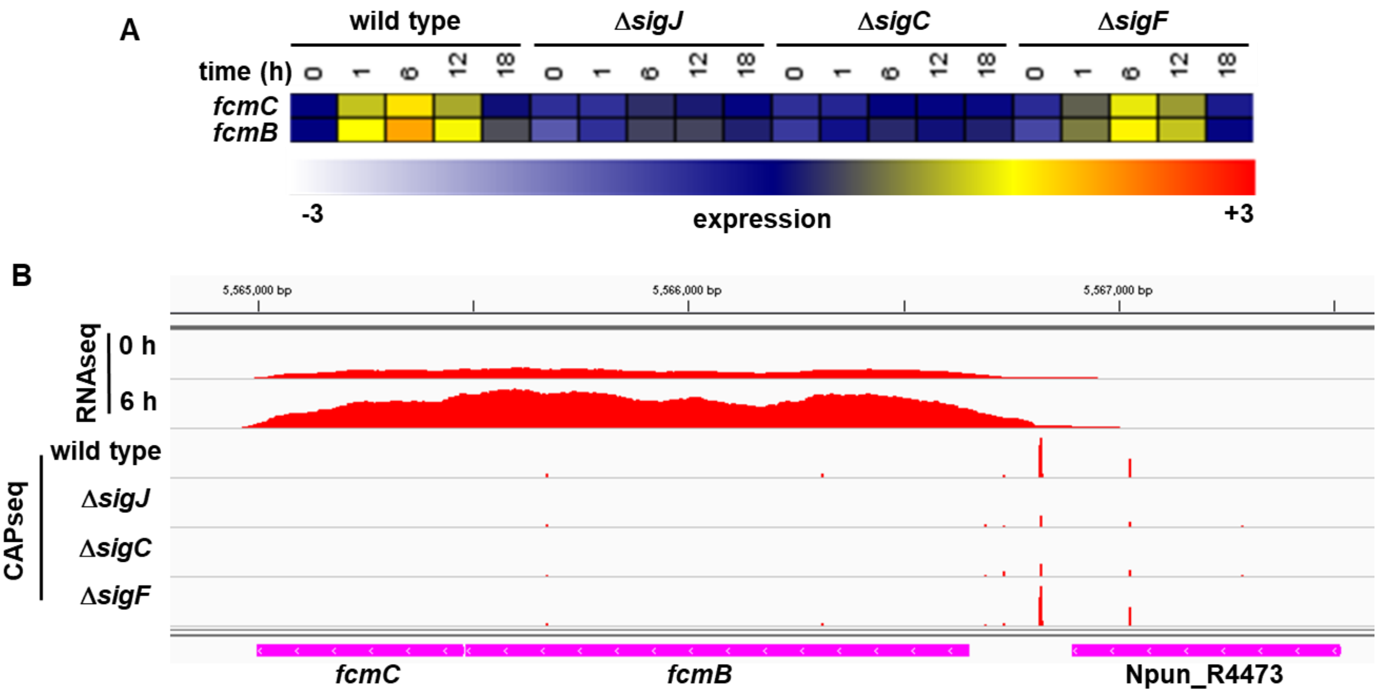

Fig. S9. Expression of fcmBC in developing hormogonia. (**A**) Heatmaps depicting RNAseq based quantification of *fcmBC* transcription in developing hormogonia (0-18h post-induction) of the wild-type strain and hormogonium sigma factor mutants, derived from (4). Expression = Experimental strain and time point/wild type t=0. (**B**) Read coverage of the *fcmBC* locus from RNAseq (4) and CAPseq (5) data sets (as indicated).

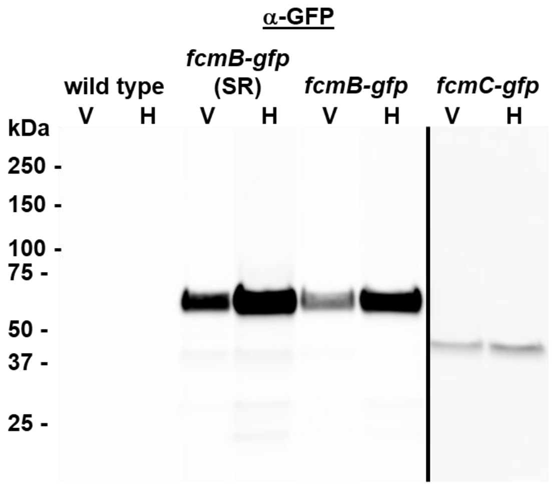

Fig. S10. Immunoblot analysis of FcmB-GFP and FcmC-GFP with α-GFP antibodies. Strains as indicated. V = vegetative filaments, H = hormogonium filaments. Note that all samples were detected on the same membrane, however, detection of FcmC-GFP required longer exposure times, and thus the black line indicates that these were from two different images with different exposure for the same membrane. The expected molecular weight of FcmB-GFP is ~69 kDa and the expected molecular weight of FcmB-GFP is ~45 kDa.

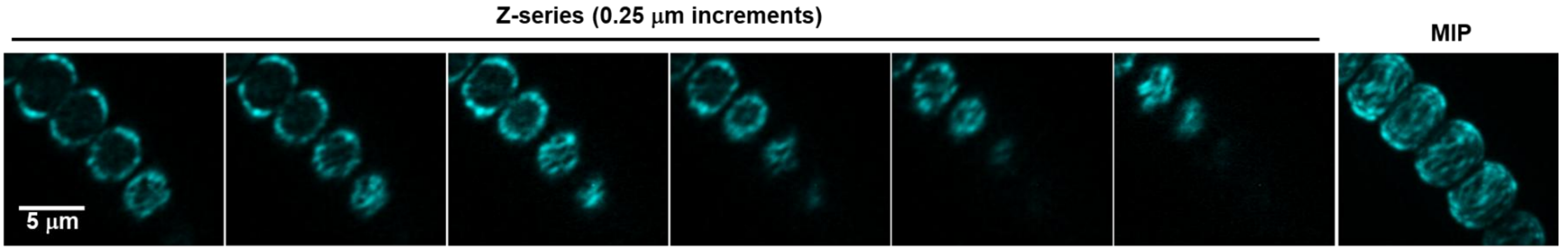

Fig. S11. Structured illumination microscopy of the *fcmB-gfp* (SR) strain. Depicted are fluorescence micrographs of FcmB-GFP (Cyan) from a section of a Z-series and the maximal intensity projection (MIP) for the Z-series, as indicated.

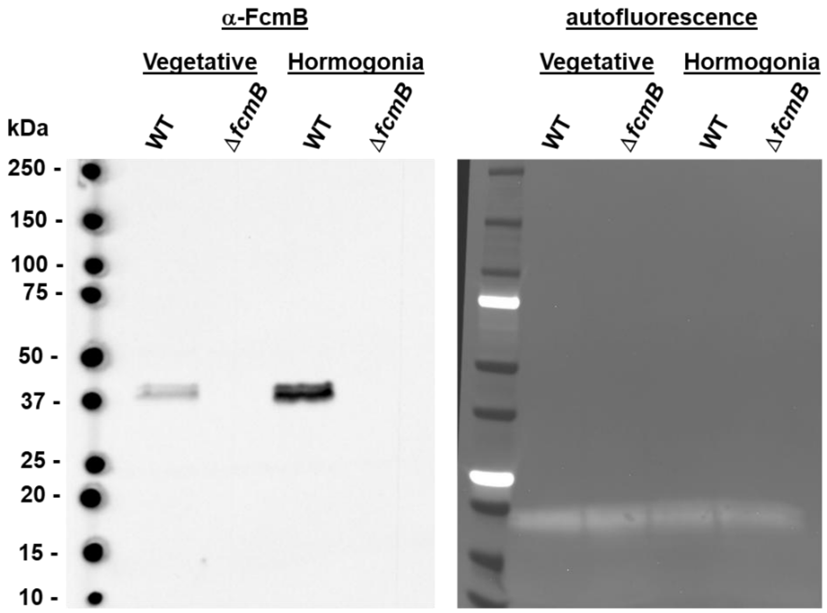

Fig. S12. Immunoblot analysis of FcmB with α-FcmB antibodies. Strains and filament types as indicated. The expected molecular weight of FcmB is 42 kDa. Autofluorescence of phycobiliproteins serves as a protein loading control.

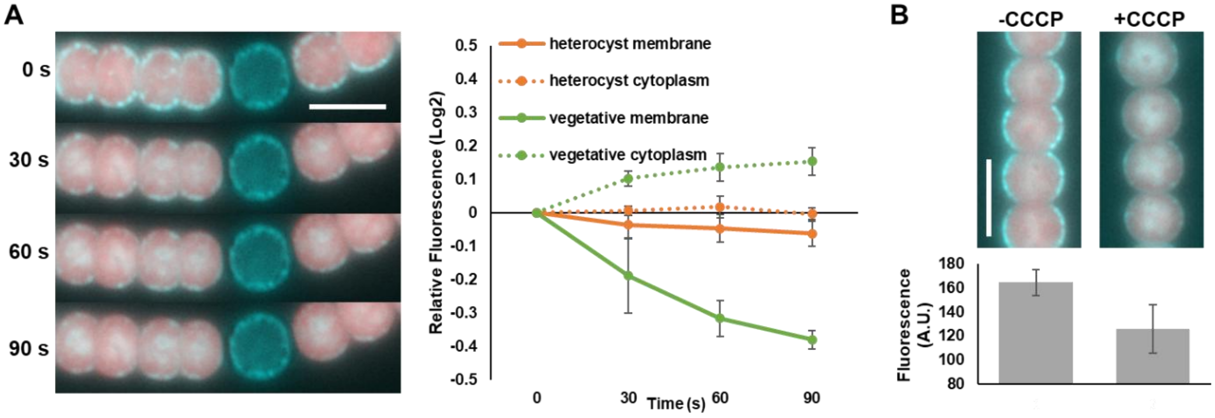

Fig. S13. Localization of FcmB-GFP in response to fluorescence imaging and CCCP treatment. (**A**) Light micrographs of time lapse fluorescence microscopy of FcmB-GFP (Cyan) and autofluorescence (Red), and quantification of membrane and cytoplasmic fluorescence in the *fcmB-gfp* (SR) strain. Representative vegetative cells and a heterocyst are depicted. Relative fluorescence derived from the ratio of fluorescence at each time point / t = 0. n = 3, error bars = +/- 1 S.D. Filaments were pre-exposed to illumination for FcmB-GFP imaging for 10 s, then imaged at 30 s intervals. White bar = 5 μm. Heterocysts can be identified by the absence of autofluorescence. (**B**) Fluorescence micrographs of FcmB-GFP (Cyan) and autofluorescence (Red) for CCCP treated and untreated cells and quantification of FcmB-GFP membrane fluorescence. Representative vegetative cells depicted. White bar = 5 μm. n = 3, error bars = +/- 1 S.D.

Table ST2. Plasmids, strains, and oligonucleotides used in this study

Strain name	Description	Source	
N. punctiforme ATCC29133	wild type	ATCC	
UOP177	Δ*rodA* (Npun_R5165)	This study	
UOP180	Δ*fcmC* (Npun_R4471)	This study	
UCCS100	Δ*fcmC* (Npun_R4472)	This study	
UCCS114	*fcmB-gfp* (SR)	This study	
UCCS116	*fcmC-gfp*	This study	
UCCS125	*fcmB-gfp*	This study	
**Plasmid name**	**Description**	**Primers used**	**Source**
pDDR470	Suicide vector for in-frame deletion of *rodA*	1-4	This study
pDDR477	Suicide vector for in-frame deletion of *fcmC*	5-8	This study
pDDR478	Suicide vector for in-frame deletion of *fcmB*	9-12	This study
pGAP111	Suicide vector for allelic replacement of *fcmC* with *fcmC-gfp*	5,8,13-14	This study
pGAP112	Suicide vector for allelic replacement of *fcmB* with *fcmB-gfp*	12,15-17	This study
pGAP102	Shuttle vector containing *fcmB* expressed from the *petE* promoter	17-18	This study
pGAP103	Shuttle vector containing *fcmC* expressed from the *petE* promoter	19-20	This study
pDDR541	Shuttle vector containing *rodA* expressed from its native promoter	21-22	This study
pPDB100	pUT18-*mreD* (Npun_R1839)	23-24	This study
pPDB101	pUT18c-*mreD* (Npun_R1839)	23-24	This study
pPDB104	pUT18-*mreC* (Npun_R1840)	25-26	This study
pPDB105	pUT18c-*mreC* (Npun_R1840)	25-26	This study
pAN100	pUT18-*mreB* (Npun_R1841)	27-28	This study
pAN101	pUT18c-*mreB* (Npun_R1841)	27-28	This study
pGMJ100	pUT18-*fcmC*	29-30	This study
pGMJ101	pUT18c-*fcmC*	29-30	This study
pGMJ102	pKT25-*fcmC*	29-30	This study
pGMJ103	pKNT25-*fcmC*	29-30	This study
pGMJ104	pUT18-*fcmB*	31-32	This study
pGMJ105	pUT18c-*fcmB*	31-32	This study
pGMJ106	pKT25-*fcmB*	31-32	This study
pGMJ107	pKNT25-*fcmB*	31-32	This study
**Primer name**	**Sequence**	**Primer Number**	
NpR5165-5'-F	atataggatccGTACGTTCCACTGAAACAG	1	
NpR5165-5'-R	caataatacttcagTAATAACATTGTGCCAATTTAGAC	2	
NpR5165-3'-F	gcacaatgttattaCAGAAGTATTATTGATTCGTC	3	
NpR5165-3'-R	atatagagctcGATAAACAGCTTGATGAATTTGC	4	
NpR4471-5'-F	ATATAGGATCCAGCTACTGACCCTTTAATGG	5	
NpR4471-5'-R	ccttaaaaatcatcTTGGAACATCAGCTTTATGC	6	
NpR4471-3'-F	ctgatgttccaaGATGATTTTTAAGGAAAAATCAGG	7	
NpR4471-3'-R	ATATAGAGCTCTTTTTTCGGCAGCTTCTATG	8	
NpR4472-5'-F	ATATAGGATCCAAGACTAGTGCAGCAATTTC	9	

NpR4472-5'-R	ctttatgccctGTCTGTCATGAAAGCTCCTAG	10
NpR4472-3'-F	catgacagacAGGGCATAAAGCTGATGTTC	11
NpR4472-3'-R	ATATAGAGCTCGACTATTAGCTCGCTGGAC	12
NpR4471-gfp-5'-R	atatacccgggAAAAATCATCGAGAAACTGACTAAG	13
NpR4471-gfp-3'-F	atataactagtGGAAAAATCAGGTTTGTGAG	14
NpR4472-gfp-5'-R	atatacccgggATGCCCTAGCAGCGCGATTG	15
NpR4472-gfp-3'-F	atataactagtAGCTGATGTTCCAATGGTC	16
NpR4472-BamHI-F	atataggatccATGACAGACCAACCTTCC	17
NpR4472-SacI-R	atatagagctcTTATGCCCTAGCAGCGCGATTG	18
NpR4471-BamHI-F	atataggatccATGTTCCAATGGTCAAAAAAGG	19
NpR4471-SacI-R	atatagagctcTTAAAAATCATCGAGAAACTGACTAAG	20
PNpR5165-BamHI-F	atataggatccGCGTGCCACTAGAGATTTC	21
NpR5165-SacI-R	atatagagctcTCAATAATACTTCTGCCTTTGG	22
NpR1839-TH-BamHI-F	atataggatcccATGAAGATTCCTGCATTTGG	23
NpR1839-TH-KpnI-R	atataggtacccgTTGCTCCAACAATTTCATTCGTTG	24
NpR1840-TH-BamHI-F	atataggatcccATGGTTACAATACGGCGCTG	25
NpR1840-TH-KpnI-R	atataggtacccgCTTAGACTTTTGCGGCAGTTG	26
NpR1841-TH-BamHI-F	atataggatcccGTGGGTATTTTTAGGAACTTTC	27
NpR1841-TH-KpnI-R	atataggtacccgCATATTGCGAGAGCTTTCG	28
NpR4471-TH-BamHI-F	atataggatcccATGTTCCAATGGTCAAAAAAGG	29
NpR4471-TH-KpnI-R	atataggtacccgAAAATCATCGAGAAACTGACTAAG	30
NpR4472-TH-BamHI-F	atataggatcccATGACAGACCAACCTTCC	31
NpR4472-TH-KpnI-R	atataggtacccgTGCCCTAGCAGCGCGATTG	32

Table ST3. Proteins used in phylogenetic analysis of FcmB

Identifier	Protein	Organism
WP_012410800.1	FcmB	*Nostoc punctiforme*
WP_044522477.1	FcmB	*Nostoc sp. PCC 7120*
WP_026734259.1	FcmB	*Fischerella sp. PCC 9605*
WP_011613886.1	FcmB	*Trichodesmium erythraeum*
WP_041565291.1	MreB	*Nostoc punctiforme*
WP_044520478.1	MreB	*Nostoc sp. PCC 7120*
WP_044136450.1	MreB	*Trichodesmium erythraeum*
WP_026735085.1	MreB	*Fischerella sp. PCC 9605*
WP_011243524.1	MreB	*Synechococcus elongatus PCC 7942*
YP_420328.1	MamK	*Magnetospirillum magneticum AMB-1*
CAM78025.1	MamK	*Magnetospirillum gryphiswaldense MSR-1*
ZP_00054405.2	MamK	*Magnetospirillum magnetotacticum MS-1*
YP_002955471.1	MamK	*Desulfovibrio magneticus RS-1*
YP_866166.1	MamK	*Magnetococcus marinus MC-1*
WP_231848947.1	MamK	*Paramagnetospirillum magneticum*
P11904.1	ParM	*Escherichia coli*
NP_941097.1	ParM	*Serratia marcescens*
NP_058228.1	ParM	*Salmonella enterica subsp. enterica serovar Typhi*
NP_863404.1	ParM	*Salmonella enterica subsp. enterica serovar Typhimurium*
AAT37581.1	ParM	*Escherichia coli*
AAL72301.1	ParM	*Shigella flexneri 2a str. 301*
BAA24871.1	AlfA	*Bacillus subtilis*
AAK79133.1	AlfA	*Clostridium acetobutylicum ATCC 824*
NP_116614.1	Actin	*Saccharomyces cerevisiae S288C*
NP_001605.1	Actin	*Homo sapiens*
NP_001014725.1	Actin	*Drosophila melanogaster*
NP_499809.1	Actin	*Caenorhabditis elegans*
XP_644247.1	Actin	*Dictyostelium discoideum AX4*
NP_420354.1	MreB	*Caulobacter vibrioides CB15*
NP_390681.2	MreB	*Bacillus subtilis subsp. subtilis str. 168*
AAA58054.1	MreB	*Escherichia coli str. K-12 substr. MG1655*
NP_228398.1	MreB	*Thermotoga maritima MSB8*
NP_781022.1	MreB	*Clostridium tetani E88*
AAF93588.1	MreB	*Vibrio cholerae O1 biovar El Tor str. N16961*
ACU27363.1	Alp7A	*Bacillus subtilis*
WP_000588477.1	FtsA	*Escherichia coli*
Q9WZU0	FtsA	*Thermotoga maritima*

Movie S1 (separate file). Time-lapse microscopy of motile hormogonia in the wild type, Δ*fcmB*, and Δ*fcmC* strains.

Data Set S1 (separate file). Precent Identity and percent positive matrix for protein sequences used to build phylogenetic tree in Fig. 1B.

SI References

1. Wang J, Chitsaz F, Derbyshire MK, Gonzales NR, Gwadz M, Lu S, Marchler GH, Song JS, Thanki N, Yamashita RA, Yang M, Zhang D, Zheng C, Lanczycki CJ, Marchler-Bauer A. 2023. The conserved domain database in 2023. Nucleic Acids Res 51:D384–D388. 10.1093/nar/gkac1096.
2. Cho YW, Gonzales A, Harwood TV, Huynh J, Hwang Y, Park JS, Trieu AQ, Italia P, Pallipuram VK, Risser DD. 2017. Dynamic localization of HmpF regulates type IV pilus activity and directional motility in the filamentous cyanobacterium *Nostoc punctiforme*. Mol Microbiol 106:252–265. 10.1111/mmi.13761 [doi].
3. Shih PM, Wu D, Latifi A, Axen SD, Fewer DP, Talla E, Calteau A, Cai F, Tandeau de Marsac N, Rippka R, Herdman M, Sivonen K, Coursin T, Laurent T, Goodwin L, Nolan M, Davenport KW, Han CS, Rubin EM, Eisen JA, Woyke T, Gugger M, Kerfeld CA. 2013. Improving the coverage of the cyanobacterial phylum using diversity-driven genome sequencing. Proc Natl Acad Sci U S A 110:1053–1058. 10.1073/pnas.1217107110 [doi].
4. Gonzalez A, Riley KW, Harwood TV, Zuniga EG, Risser DD. 2019. A Tripartite, Hierarchical Sigma Factor Cascade Promotes Hormogonium Development in the Filamentous Cyanobacterium *Nostoc punctiforme*. mSphere 4:10.1128/mSphere.00231–19. e00231-19 [pii].
5. Harwood TV, Risser DD. 2021. The primary transcriptome of hormogonia from a filamentous cyanobacterium defined by cappable-seq. Microbiology (Society for General Microbiology) 167:. 10.1099/mic.0.001111.
